# Bibliographical Notices, &c.

**Published:** 1846-10-01

**Authors:** 


### Bibliographical Notices.

---

The Harveian Oration, delivered before the Royal College of Physicians, London, June 1846. By *John Elliotson,* M.D. F.R.S. &c. With an English Version and Notes. 8vo. pp. 70. London. Baillere.

The greater part of this oration is occupied with bald and profitless tales about Linacre, Caius, and other old fellows of the College, and with a dreary account of the disputes about the circulation of the blood in Harvey's time. The narrative is, however, enlivened with random remarks, some of which are really very amusing. For example, four of the silly opposers of Harvey's views are thus summarily consigned to rest:—*requiescunt in pace Simon Boullotius cum Hugone Chaslesio; Franciscus quoque Bazin cum Philippo Hardouino suo,*

"—— not a pinch of dust remains of Cheops."

*Sanguis autem illum suum " motum circularem" etiam nunc improbus tenet.* The classicality of the Latin (*illum suum* is surely Elliotsonian) is strangely set off by the English quotation. By the bye, Sir Astley Cooper is made, *nolens volens,* to speak in Latin—a feat which, we dare say, the worthy Baronet could not well do in life: *solebut in prœlectionibus chirurgicis jactantius aliquanto quotannis affirmare, " se, Deo gratias, nihil de medicinâ scire;" quanquam ipse compluria millia sterlingorum singulis annis faceret, quum iis morbis, qui omnino medicorum essent, prœscriberet.*

Here is a curious phrenological conjecture: " Harvey's brain must have been of superior composition and organization to those of Linacre and Caius, for he was educated precisely as they had been, was an excellent classic, writing Latin fluently and elegantly, and enjoying the ancient poets to absolute rapture, and well versed in Aristotle and Galen. Yet he found no pleasure in books as books, but only as the depositaries of truth and genius, and therefore never rested without testing the contents of scientific works, however reverenced among the learned, by a reference to nature herself, of whom they profess to be the delineators."

Dr. Elliotson is justly indignant that no public honour was conferred upon

Harvey during his life-time: in this respect he was "like the other greatest names in our profession—Glisson, Sydenham, Jenner, John and William Hunter. Indeed the majority of really great British medical men have received no titles; and the majority of those who have received them have been distinguished for nothing but good luck, and often but for little even of that."

The allusion to Mesmerism is made with all the coyness of a shame-faced lover. Dr. E. will not speak out the name of his inamorata; he only leaves us to guess who she is in the following passage, which forms the peroration of his speech:

"A body of facts is presented to us not only wonderful in physiology and pathology, but of the very highest importance in the prevention of suffering under the hands of the surgeon and in the cure of disease. The chief phenomena are indisputable: authors of all periods record them, and we all ourselves witness them, some rarely, some every day. The point to be determined is whether they may be produced artificially and subjected to our control: and it can be determined by experience only. The loss of common feeling,—anæsthesia, is but a form of palsy, and in it wounds give no pain. If this condition can be induced temporarily by art, we of necessity enable persons to undergo surgical operations without suffering. Whether the artificial production of those phenomena, or the performance of the processes which so often induce them, will mitigate or cure disease, can likewise be determined by experience only. It is the imperative, the solemn, duty of the profession, anxiously and dispassionately to determine these points by experiment, each man for himself. I have done so for ten years, and fearlessly declare that the phenomena, the prevention of pain under surgical operations, the production of repose and comfort in disease, and the cure of many diseases, even after the failure of all ordinary means, are true. In the name, therefore, of the love of truth, in the name of the dignity of our profession, in the name of the good of all mankind, I implore you carefully to investigate this important sbuject."

We regret that Dr. E. has most injudiciously made this Harveian Oration the medium of again proclaiming to the world his belief in the materialism of the soul. Would that he did but follow his own advice and become as a little child in the study of those momentous truths, the right appreciation of which might have kept him from the unfortunate delusions which have beset his path!

---

Mesmerism in India, and its Practical Application in Surgery and Medicine. By *James Esdaile*, M.D., Civil Assistant-Surgeon H.C.S., Bengal. 12mo, pp. 286. Longman, 1846.

We have in a preceding article (p. 506) stated the reason why any amount of curative power which mesmerism may possess is not available to the profession; and we trust we shall not be deemed inconsistent in stating a case in which it may be resorted to. Most will acknowledge that, in certain individuals, a more or less prolonged insensibility may be induced by mesmeric procedures, and it is obvious that the fact of this being so complete as to admit of the performance of surgical operations during its existence, and of surgeons availing themselves of such suspension of sensibility, says nothing *pro* or *con* concerning the remedial agency or the ultra pretensions of mesmerism. The same state has been induced by other means, such as injuries to the head, narcotics, and intoxication, during which unfelt operations have been completed. The questions then we have to decide are whether mesmerism can induce such sufficient insensibility, and whether the patient's eventually well-doing is at all compromised by the operation being undertaken under such circumstances. Dr. Esdaile adduces 73 instances of painless operations of varying degree of severity, from the introduction of a seton to the amputation of an arm or the tearing of a toe-nail, performed upon subjects who could have no inducement, and scarcely intelligence enough, to enter into any plan of collusion, in the presence of very numerous witnesses. Without agreeing with the author in the general favorable estimate he gives of mesmerism, we may state that we believe the cases we have alluded to are entitled to our belief, and that the subject is one of such vast importance as to call for a searching investigation by the authorities at home. This claim of mesmerism admits of easy testing, and we repeat if its validity is proved, that of the various monstrous pretensions of this art will not be brought in anywise nearer admission, while a vast boon will have been secured to suffering mankind.

---

I. Fever Physiologically considered. By *David M'Connell Reed,* Esq. Octavo, pp. 260. Churchill, 1846.

II. Annual Report of the Cork Fever Hospital for the Year 1845.

III. Medical Report of the House of Recovery and Fever Hospital, Cork Street, Dublin. By *George Kennedy,* M.D. M.R.I.A. Dublin, 1846.

Dr. Reed's work conveys little useful information that we can discover upon the inexhaustible and interesting subject of fever. He denies the existence of essential or idiopathic fevers, maintaining that all the symptoms are referrible to the deranged condition of certain organs, and according as they emanate from one or other of these we have Pulmonary, Cerebral, Gastric, Pleuritic, Fever, &c. The author, in the course of his work, more than once offers up hearty thanks to God for the completion of various portions of it: and as we had to read page after page from such very accessible authorities as Hooper and Thomas, we felt no less grateful when the perusal was completed.

The other two publications relate to Fever in Ireland; a subject fraught with interest when famine is impending over that unhappy land. We have nothing to do with politics in this Journal, but we should be wanting in proper feeling if we did not offer our humble tribute of approbation to the earnest endeavours the late and present ministry have employed to ward off fever, that desolating scourge of Ireland, by a due supply of food. It was a noble reply of Sir Robert Peel, when taunted with being too precipitate (alas! who will *now* say so?) in his arrangements for this purpose—"Am I to wait and see how much flux, diarrhœa, and fever a people can bear before ministering to their necessities?"

In the Report of the Cork Epidemic we find the Reporter quoting Hippocrates to the Governors in illustration of a *green appearance of the tongue,* noticed by the Greek Physician, but not by the moderns until Dr. Beamish published an account of it in the *3rd vol. of the Trans. Coll. Phys., Ireland.* Dr. B. has noticed it altogether in 86 cases, 24 of which have occurred during this epidemic. The prognosis in such cases is however favorable, contrary to the statement of Hippocrâtes, for 83 of the patients recovered.

Dr. Kennedy's Report is a very able one, containing much valuable information on the various forms of fever; but too condensed to admit of analysis.

---

On the Antidotal Treatment of the Epidemic Cholera. By *John Parkin,* M.D., &c. Octavo, pp. 60. London, 1846.

Our only motive for noticing this pamphlet is to protest against the very improper use, which the author has made of the Medico-Chirurgical Review to puff off his strange production. We were not a little surprised to observe that the advertisements in the newspapers and elsewhere of this most uncalled-for new edition of the "Antidotal Treatment" were backed with the approval of this Journal; thus: "Carbonic acid is an antidote."—*Med. Chir. Review.*

The notice, from which this disconnected scrap is so adroitly taken, was written by the late Dr. Johnson in his usual playful mood, when disposed to ridicule some absurd work that came under his notice. So it was on the present occasion, as the following passage will shew:—

"Mr. Parkin is satisfied that carbon or carbonic acid is an antidote for the poison of cholera. He has told the world so several times already; but, as it won't believe him, he has written a book. If that fails to convince, he must adopt the expedient of my uncle Toby, and tell this generation to "go and be d———."—*Med. Chir. Review, No.* 50, *Oct.* 1836.

We trust that Dr. P. will not repeat his scarcely-honest procedure.

---

Observations on the Edinburgh Pharmacopœia, and on the Dispensatories of Dr. Christison and Dr. A. T. Thomson; to which are added Illustrations of the Present State of Pharmacy in England. By *Richard Phillips,* F.R.S. L. & E. &c. London: Highley, 1846.

In a recent number of the British Quarterly, it was well observed that men of science might be divided into two classes—those who seek after truth, and those who avoid error. So also may literary men be classified, and form two as distinct orders—those who write, and those who comment on what is written; the one being fully as useful as the other. The work now under consideration comes under the latter class, and is well calculated to uphold the value and importance of it.

It is evidently a "work of love," as nothing else could compensate for the great labour and time bestowed on such a careful analysis of the works reviewed. Indeed, there are few men so competent as Mr. Phillips for the task he has undertaken, and notwithstanding he has the reputation of using the knife boldly, still he rarely comes into contact with the sound parts, though at times cutting very close to them. Were we disposed to criticise as minutely as he has done in a few instances, we might perhaps place our finger on a tender place or two.

We have gone through the objections he takes, and several of the calculations he makes in support of them, and have found them to be perfectly correct. The errors which the author has exposed are of various degrees of importance, and some of them exhibit a degree of carelessness which is almost incredible. Had there been space, we should have adduced an example or two.

In the latter part of the Pamphlet, there are some very forcible "Illustrations of the present State of Pharmacy in England," which is a matter more directly concerning the public than even the great errors of the works reviewed. We fear much that the points selected for illustration are but very few compared with the mass, which would present itself were the enquiry continued. No doubt many err through ignorance, and many through knavery: the former should be removed, and the latter punished. If the Charter of the Pharmaceutical Society is based upon the principle of reform and improvement in its members, they must not tarry on their road; for verily, even by this sketch of their failings, they have some heavy work cut out for them. Our author says, he only leaves his post for a season; with the promise to return to it, if necessary, when sufficient time may be supposed to have elapsed for remedying the existing delinquencies, whether arising from carelessness, ignorance, or fraud.

---

A Medical Topography of Tunbridge Wells. By *Robert Hutchinson Powel,* M.B., M.D. 12mo, pp. 176. Tunbridge, 1846.

It is well that medical practitioners should bear in mind that the railway brings within an easy distance a very efficacious chalybeate. There is a large class of cases which this is likely to benefit. Dr. Powel has prepared an interesting little work, describing the locality, detailing opinions upon the curative powers of the waters, and indicating the cases likely to be benefited by their use. As his work is intended as much for the public as the profession, we think he enters into more medical details than are desirable. It is strange that a spot, possessing so many natural advantages, should have been so often comparatively neglected, and we hope this little *brochure* will have the effect of calling attention to it. We have, however, a question to put to Dr. Powel, namely, how comes it that children, who have been once to Tunbridge, have so frequently a marked objection to return? It is an interest that must be conciliated.

### Quain on the Arteries.

Our readers will observe, among the Advertisements, the announcement of a great reduction in the price of this valuable work. It is an honour to the age and country, and we are much pleased to find it thus brought within the means of private individuals, as well as public institutions.

